# Diaphragmatic Castleman's disease: A rare lymphoproliferative disorder: Clinical and radiological perspectives

**DOI:** 10.1016/j.radcr.2024.09.077

**Published:** 2024-09-25

**Authors:** Pritish Aher, Raed Zughul, Sameer Samtani, Sarv Priya, Yoel Siegel, Chris Schettino

**Affiliations:** a Radiology, University of Miami Miller School of Medicine, Miami, FL, USA; b Radiology, University of Iowa Hospitals and Clinics, Iowa City, IA, USA

**Keywords:** Diaphragmatic mass, Thoracic liposarcoma, Cardiophrenic mass, Thoracic Castleman disease, Lymphoproliferative disorders

## Abstract

Castleman's disease (CD) is a rare, benign nonclonal lymphoproliferative disorder with an unclear etiology, presenting significant diagnostic challenges due to its nonspecific features. CD is categorized into unicentric (UCD) and multicentric (MCD) types, with MCD further divided into HHV-8-associated and idiopathic (iMCD) forms. Clinical manifestations include fever, weight loss, night sweats, and organomegaly, with specific symptoms depending on the subtype. Diagnostic criteria for CD involve a combination of major criteria-histopathologic examination and minor criteria. Imaging techniques, including CT, MRI, and PET-CT, play a crucial role in diagnosis, staging, and differentiation from other diseases. This paper discusses the pathophysiology, clinical features, diagnostic criteria, and imaging findings of CD, illustrated by a case of a patient with renal disease with incidentally detected a right cardiophrenic mass. The case highlights the importance of comprehensive imaging and clinical evaluation in managing CD.

## Case presentation

A 30-year-old male presented to the emergency department with anasarca, a history of renal disease, and hypertension. Originally from Colombia, he was diagnosed with minimal change disease and mesangial IgA deposition, indicating IgA nephropathy, and had been treated with pulse steroids and dialysis. He reported worsening lower extremity edema but no shortness of breath, chest pain, nausea, vomiting, or abdominal pain. On physical examination, his head, ears, nose, and throat were unremarkable. Eyes showed no signs of conjunctival erythema, and his sclerae were nonicteric. His neck showed no jugular venous distention. Cardiovascular examination revealed a regular rate and rhythm without murmurs or rubs. His lungs were clear to auscultation bilaterally. The abdomen was soft, nontender, and nondistended. Neurologically, the patient was alert, awake, and oriented to person, place, and time, with no focal deficits. His extremities showed edema, and pulses were strong. There were no rashes. Laboratory findings indicated elevated blood urea nitrogen and creatinine levels, decreased serum protein, and decreased calcium levels. Patient's HIV and HHV tests were negative. The patient was admitted for evaluation of renal disease.

**Fig. 1:**
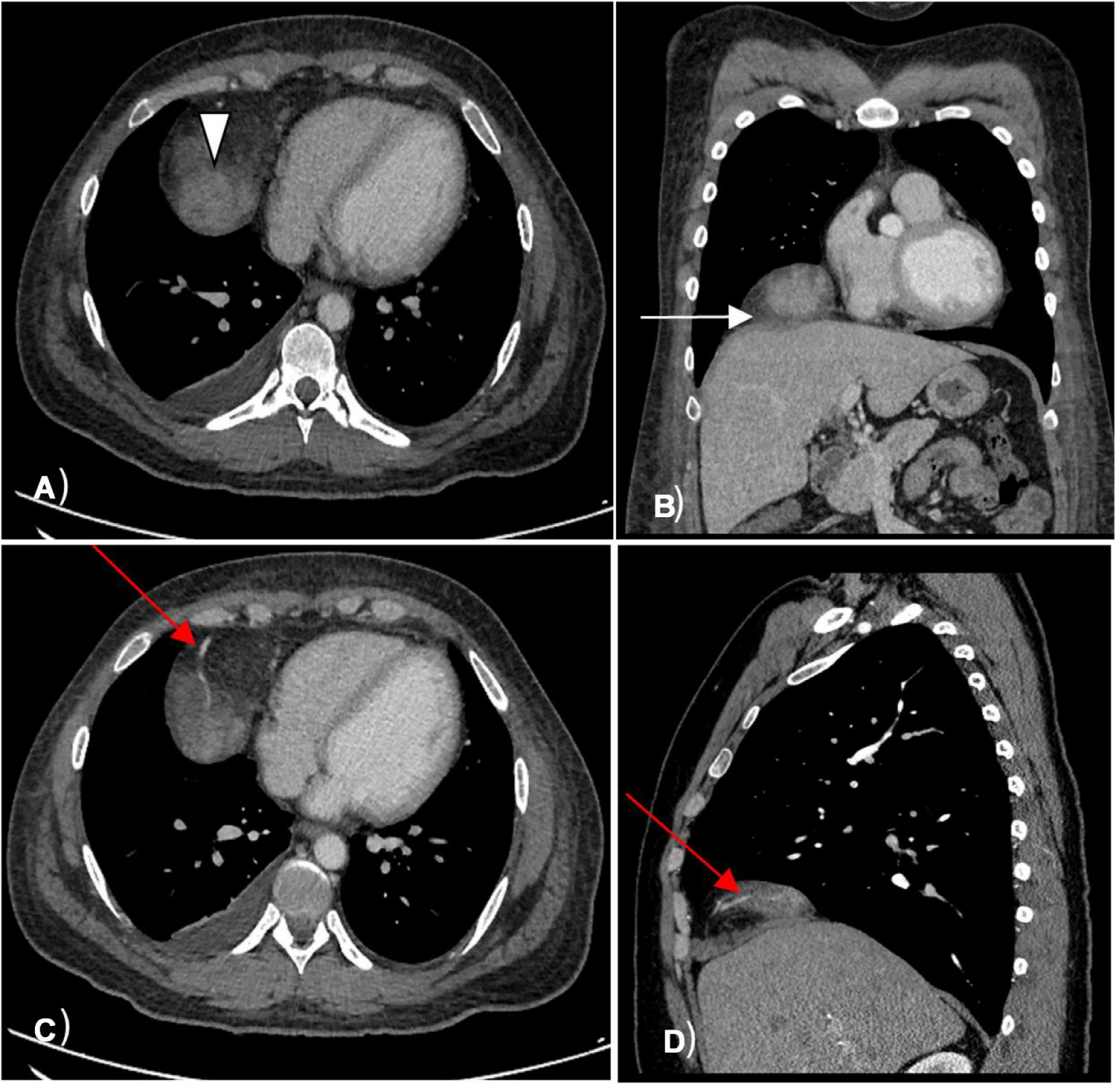
Axial CT image of the lower chest (A) demonstrates soft tissue mass in the right cardiophrenic recess (white arrowhead); Coronal CT image of the chest (B) demonstrates infiltration of the right hemidiaphragm by the mass (white arrow); Axial CT image of the lower chest (C) demonstrates a feeding vessel into mass (red arrow) that is seen as well in the sagital CT image of the chest (D).

**Fig. 2:**
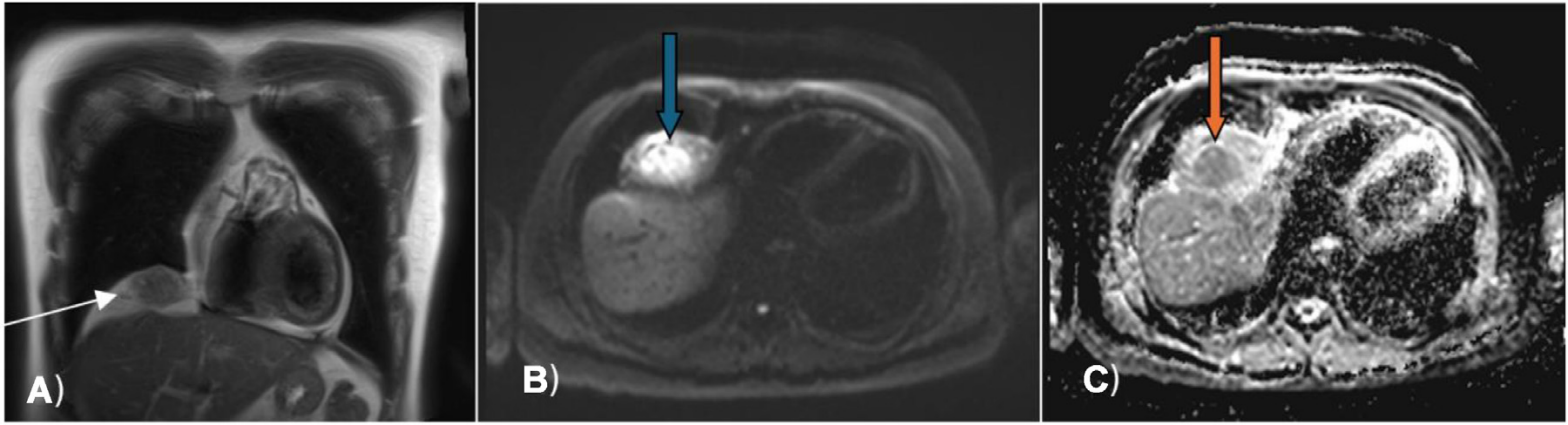
Coronal T2 weighted MR image of the chest (A) demonstrates heterogeneous signal intensity within the mass (white arrow). Blue arrow in the diffusion weighted MR image (B) points to the increased signal intensity within the mass; and corresponding low signal intensity (orange arrow) on the ADC map image (C).

**Fig. 3:**
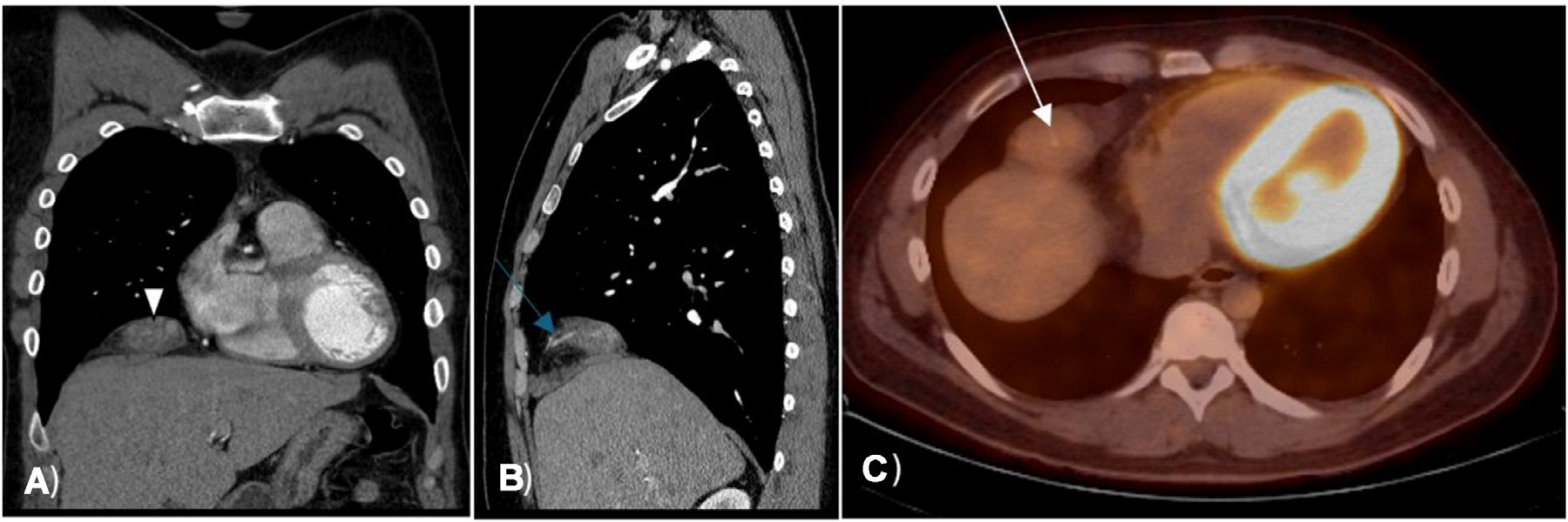
Coronal (A) and sagittal (B) CT images of the chest demonstrate decrease in size of the mass 4 months after later (white arrowhead) with the feeding vessel again illustrated. Axial PET/CT image of the lower chest (C) taken 3 months after first encounter demonstrates mild FDG uptake within the mass (white arrow).

Radiological evaluation with a CT chest revealed an incidentally detected ill-defined, heterogeneously enhancing mass in the right cardiophrenic recess with a feeding vessel passing through it (Fig. 1). The initial differential diagnosis included arteriovenous malformation due to the presence of a feeding vessel. For better characterization, a chest MRI was performed, revealing a T1 hypointense and T2 hyperintense mass with heterogeneous intense enhancement on a postcontrast study. The diffusion-weighted MRI showed increased signal intensity within the mass and corresponding low signal intensity on the ADC map image, with additional differential diagnosis of liposarcoma or rhabdosarcoma was given (Fig. 2). Follow-up PET CT scan indicated a mixed-density mass with increased FDG uptake (SUV max of 3.8) in the right cardiophrenic region, concerning for liposarcoma, with additional considerations of a fibrous tumor due to the FDG uptake and heterogeneous appearance from hemorrhage or necrosis within the lesion (Figs. 1 and 3).

The patient underwent resection of the right diaphragmatic mass with diaphragmatic reconstruction. Intra-operative frozen section analysis suggested a lymphoid-rich neoplasm, likely thymoma. However, the final histopathological diagnosis of the resected specimen revealed reactive lymphoid hyperplasia with associated reactive neo-vascularization and prominent hyalinization with an onion-skin appearance, consistent with unicentric, vascular hyaline type Castleman disease.

## Discussion

Castleman disease is a lymphoproliferative disorder, the cause of which remains unknown. It can manifest in various parts of the body, including the neck, chest, abdomen, and pelvis. CD can present as benign lesions. Pathologically, it is classified into hyaline vascular type (HV-CD), plasma cell type, mixed type, human herpesvirus (HHV)-8 associated Castleman disease. The disease is clinically categorized as either UCD, localized or MCD, multifocal. The hyaline-vascular variant is more prevalent than the plasma-cell type, accounting for 91% of total cases. The incidence of UCD is approximately 16 per million patient/year and affects individuals of all age groups.

The incidence of iMCD is estimated to be around 5 per million patient/year [1,2]. Most documented occurrences of thoracic CD have been typically identified as focal mediastinal masses on chest radiographs or computed tomography scans. However, several cases have been reported, where the nodal mass has invaded adjacent structures or has presented as multiple enlarged lymph nodes [2]. CD presents a considerable challenge in clinical practice due to the lack of specific features that distinguish it from other conditions causing lymphadenopathies [1,2]. Diagnostic criteria for CD involve a combination of major criteria-histopathologic examination, lymph node enlargement-and minor criteria laboratory abnormalities such as elevated CRP/ESR, anemia, and hypoalbuminemia. Therefore, confirmation of the diagnosis typically requires the patient to meet both major criteria, at least 2 minor criteria [3].

CD is a rare benign lymphoproliferative disorder of unknown cause. It can appear in the neck, chest, abdomen, and pelvis. CD is pathologically classified into hyaline vascular type (HV-CD), plasma cell type, mixed type, and HHV-8 associated. The exact cause is unknown, but impaired immunoregulation, leading to excessive B lymphocyte and plasma cell proliferation, is suspected. This can result in chronic inflammation, viral infections, and abnormal cytokine modulation. CD is closely associated with HIV, particularly HHV-8 in MCD cases. iMCD has no known cause. Symptoms include fever, weight loss, night sweats, fatigue, splenomegaly or hepatomegaly, anasarca, and pleural effusion [4]. Diagnosis requires meeting both major criteria-histopathologic examination to determine node involvement, excluding other disorders, and presence of enlarged lymph nodes and at least 2 minor laboratory criteria, including elevated CRP or ESR, anemia, thrombocytopenia or thrombocytosis, hypoalbuminemia, renal dysfunction or proteinuria, and polyclonal hypergammaglobulinemia [2,3,5].

The causes of Castleman disease vary by subtype. UCD may originate from neoplastic stromal cells, such as follicular dendritic cells. MCD associated with HHV-8 is driven by uncontrolled HHV-8 infection, while the causes of HHV-8-negative or idiopathic multicentric Castleman disease is less clear, with IL-6 involvement in some cases. In POEMS-associated CD, monoclonal plasma cells cause excessive VEGF and IL-12 production due to somatic mutations. Staging, including serum protein electrophoresis, bone marrow examination, and imaging, is crucial before treatment to assess disease extent and subtype. Diagnosis starts with a lymph node biopsy, with 2017 criteria defining iMCD histopathological features as hypervascular, plasmacytic, or mixed types [[2], [3], [4], [5]].

Radiological investigations reveal varied features of CD. Thoracic CD often presents as a solitary mediastinal or hilar mass but can also appear in the pleura, pericardium, intercostal space, or lung. Pleural CD might show as an interlobar mass or massive pleural effusion, pericardial disease as a cystic structure, and intercostal disease as an extra thoracic mass with rib erosion. Intrapulmonary CD typically appears as a solitary lung mass, with rare coarse or branchlike calcifications. Multicentric CD usually presents with bilateral hilar and mediastinal lymphadenopathy, diffuse pulmonary infiltrations, hepatosplenomegaly, and ascites [3,4,6]. On CT, thoracic UCD often appears as a hypervascular, homogenously enhancing mass or conglomerate mass involving contiguous structures. It may also present as matted lymphadenopathy in a single mediastinal compartment. MCD typically shows bilateral hilar and mediastinal lymphadenopathy, centrilobular nodular opacities, and occasionally less common airspace or airway involvement. Intense contrast enhancement indicates hypervascularity, and prominent feeding vessels, though variable, can aid in surgical planning. Larger lesions may exhibit a heterogeneous appearance due to fibrosis, necrosis, and intratumoral hypodensity, while intranodal calcifications can appear coarse or branching [4,6,7]. MRI is useful in Castleman disease for visualizing mass relationships with adjacent structures and highlighting feeding vessels, which may appear as flow voids on T2-weighted images. On T1-weighted images, masses or affected lymph nodes usually appear hypointense to isointense relative to skeletal muscle, and isointense to hyperintense on T2-weighted images. Contrast-enhanced MRI reveals homogeneous or heterogeneous enhancement, with enhancement continuing into the portal venous phase, and masses often show restricted diffusion [6]. PET-CT helps identify optimal biopsy sites, assess disease extent, and monitor progression. CD lymph nodes typically show mild to moderate hypermetabolism with a mean SUVmax of 5.6-5.8. Increased splenic activity may also be observed. While SUVmax can vary between UCD and MCD and align with active symptoms, CD lymph nodes generally have a lower median SUVmax compared to mimicking conditions. Elevated SUVmax in lesions may indicate alternative diagnoses, such as high-grade lymphoma. In POEMS syndrome, osseous lesions may also be FDG-avid [7,8].

The imaging findings for this case of renal disease reveal a right cardiophrenic infiltrative, intensely enhancing mass with feeding vessels diagnosed on CT Chest. MRI shows the mass infiltrating the right diaphragm near the cardiophrenic angle, appearing T1 hypointense and T2 hyperintense with heterogeneous enhancement. PET imaging indicates the mass is FDG avid, hypermetabolic, and heterogeneous with a maximum standardized uptake value of 3.8.

## Conclusions

CD is a rare benign lymphoproliferative disorder with unknown cause, presenting as single or multiple lesions in various body regions. While its exact etiology remains unclear, it is associated with impaired immunoregulation and increased susceptibility to viral infections including the HIV subtype associated with HHV-8. Radiological evaluation is crucial for diagnosis, with CT, MRI, and PET imaging aiding in characterization and management. In this case, imaging findings suggestive of CD included an irregular, enhancing mass with feeding vessels on CT, hyperintensity on T2-weighted MRI, and hypermetabolism on PET imaging.

In conclusion, we present a case of multimodality imaging an unusual cardiophrenic mass. While our initial concerns were centered around more ominous pathologies such as liposarcoma, the final pathologic diagnosis revealed diaphragmatic Castleman disease.

## Patient consent

Patients consent is obtained for manuscript entitled ‘Diaphragmatic Castleman's Disease: A Rare Lymphoproliferative Disorder – Clinical and Radiological Perspective’. The data used in our study is anonymized and no personal identifiers are included that could lead to the identification of individual.
